# Prenatal Exposure to Sodium Arsenite Alters Placental Glucose 1, 3, and 4 Transporters in Balb/c Mice

**DOI:** 10.1155/2015/175025

**Published:** 2015-08-03

**Authors:** Daniela Sarahí Gutiérrez-Torres, Carmen González-Horta, Luz María Del Razo, Rocío Infante-Ramírez, Ernesto Ramos-Martínez, Margarita Levario-Carrillo, Blanca Sánchez-Ramírez

**Affiliations:** ^1^Programa de Maestría en Ciencias en Biotecnología, Facultad de Ciencias Químicas, Universidad Autónoma de Chihuahua, Circuito No. 1 Nuevo Campus Universitario, 31125 Chihuahua, CHIH, Mexico; ^2^Departamento de Toxicología, Centro de Investigación y Estudios Avanzados del Instituto Politécnico Nacional (CINVESTAV), Avenida Instituto Politécnico Nacional 2508, 07360 Mexico, DF, Mexico; ^3^Departamento de Anatomía Patológica del Hospital CIMA, Avenida Hacienda del Valle No. 7120, 31217 Chihuahua, CHIH, Mexico; ^4^Laboratorio de Embriología, Facultad de Medicina, Universidad Autónoma de Chihuahua, Nuevo Campus Universitario, 31125 Chihuahua, CHIH, Mexico

## Abstract

Inorganic arsenic (iAs) exposure induces a decrease in glucose type 4 transporter (GLUT4) expression on the adipocyte membrane, which may be related to premature births and low birth weight infants in women exposed to iAs at reproductive age. The aim of this study was to analyze the effect of sodium arsenite (NaAsO_2_) exposure on GLUT1, GLUT3, and GLUT4 protein expression and on placental morphology. Female Balb/c mice (*n* = 15) were exposed to 0, 12, and 20 ppm of NaAsO_2_ in drinking water from 8th to 18th day of gestation. Morphological changes and GLUT1, GLUT3, and GLUT4 expression were evaluated in placentas by immunohistochemical and image analysis and correlated with iAs and arsenical species concentration, which were quantified by atomic absorption spectroscopy. NaAsO_2_ exposure induced a significant decrease in fetal and placental weight (*P* < 0.01) and increases in infarctions and vascular congestion. Whereas GLUT1 expression was unchanged in placentas from exposed group, GLUT3 expression was found increased. In contrast, GLUT4 expression was significantly lower (*P* < 0.05) in placentas from females exposed to 12 ppm. The decrease in placental GLUT4 expression might affect the provision of adequate fetal nutrition and explain the low fetal weight observed in the exposed groups.

## 1. Introduction

Inorganic arsenic (iAs) is a ubiquitous element and its toxicity has been demonstrated both in humans [1–5] and in experimental models [6]. Groundwater concentration of As has been documented in the literature, which reveals a very large range from less than 0.001 to 5 ppm covering natural As contamination found in more than 70 countries [7, 8]. Chronic exposure to iAs through contaminated water has been associated with reproductive disorders. Exposure has caused spontaneous abortions, stillbirths, premature births, and low birth weight infants in women of reproductive age [9–12]. The mechanisms by which iAs negatively affects reproductive health are, however, poorly understood. During pregnancy, the placenta maintains the fetal development, ensuring an adequate supply of nutrients and the removal of waste products from the fetal circulation to maternal circulation [13, 14]. Transplacental transport of nutrients is carried out by various proteins such as glucose transporters (GLUT) located in the cell membranes of maternal and fetal structures [15, 16]. To date, there are reports of the expression of the isoforms GLUT1, GLUT3, and GLUT4 in placental tissue from both humans and mice and suggestions for the function of each isoform [17]. GLUT1 has been related to the transfer of glucose from maternal circulation to the placenta. In contrast, GLUT3 seems to function in transferring glucose from the placenta to fetal blood, and GLUT4 contributes to meeting the metabolic needs of the placenta [18, 19]. Although the placenta is highly selective when preventing the passage of toxic substances to the fetus, a relationship between the levels of iAs and its metabolites found in placenta and umbilical cord blood has been reported, indicating a considerable transfer of As from the mother to the developing fetus [20]. Transplacental exposure to arsenicals may cause alterations in fetal development that leave the individual predisposed to diseases in adulthood such as atherosclerosis, type 2 diabetes mellitus and metabolic syndrome, cardiovascular disease, neuropathy, and cancer [6, 21–24]. Moreover, chronic iAs exposure has a deleterious effect on peripheral glucoregulation. It can decrease both expression and secretion of insulin in the body [25], the translocation of GLUT4 toward the surface of the membrane in adipose tissue cells [26], and glucose uptake so that glucose levels are increased in peripheral blood.

iAs is extensively metabolized by humans and many other species to yield two major methylated metabolites, methyl As (MAs) and dimethyl As (DMAs) [27]. Because the action of iAs as a toxin is fundamentally influenced by its metabolism, placental patterns of iAs and its metabolites are relevant to assessing the risk of toxicity by this metalloid.

The aim of this study was to analyze the expression of GLUT1, GLUT3, and GLUT4 transporters in placentas from mice exposed to 0, 12, and 20 ppm of sodium arsenite (NaAsO_2_) from the 8th to 18th day of gestation. Additionally, we conducted a histopathology study in the three zones of the placenta (decidua basalis, junction zone, and labyrinth) to describe the lesions and their relationship with iAs-exposure.

## 2. Materials and Methods

We obtained acetone, ethanol, methanol, potassium chloride, potassium phosphate monobasic, sodium chloride, sodium hydroxide, sodium phosphate dibasic, disodium hydrogen arsenate heptahydrate, phosphoric acid (Ultrex II), and xylene from JT Baker (Estado de México, México). Tris–HCl was purchased from Gibco BRL (Rockville, Maryland, USA) and monomethylarsenate from Supelco (St. Louis, Missouri, USA). 3-Aminopropyl triethoxysilane, hydrogen peroxide, paraformaldehyde, polyoxyethylene sorbitan monolaurate (Tween 20), dimethylarsonic acid, sodium arsenite (NaAsO_2_), and sodium borohydride were purchased from Sigma-Aldrich (St. Louis, Missouri, USA). Entellan mounting resin and sodium hydroxide were obtained from Merck (Darmstadt, Germany). For the immunohistochemistry (IHC) analysis, polyclonal anti-human GLUT1, GLUT3, and GLUT4 antibodies were purchased from Santa Cruz Biotechnology, Inc. (Santa Cruz, California, USA). A Histostain-plus kit and hematoxylin were obtained from Zymed Lab., Inc. (San Francisco, California, USA). Mice were fed pellets of Mouse Diet 5015 fish meal free from LabDiet, Purina Mills, Inc. (Richmond, Indiana, USA).

### 2.1. Animals and Treatment Conditions

Briefly, Balb/c adult female mice with a body weight of 20 g (*n* = 15) were obtained from the Bioterium of the Faculty of Chemical Sciences (Autonomous University of Chihuahua). The Institutional Animal Care and Use Committee approved all animal procedures, in compliance with the Mexican Official Technical specifications for the production, care, and use of laboratory animals [28]. Animals were randomly assigned to one of three groups and treated with 0, 12, or 20 ppm of NaAsO_2_ (concentration of the salt) dissolved in deionized water. NaAsO_2_ concentrations were selected according to the lowest observed adverse effect level (LOAEL) of 5 ppm, which had a slight decrease in the median life span and no effect on growth [29]. The estrous cycle was monitored by vaginal examination; females in estrous phase were mated with males (5 : 1) for a period of 48 hr. The following day was considered to be day 1 of gestation. Females were observed daily during and after exposure, and body weight and water consumption were also recorded. On the 8th day of gestation, the pregnant females began to receive the treatment orally* ad libitum *until the 18th gestation day. The control group (0 ppm) was given only deionized water. At the end of treatment, the animals were euthanized by asphyxiation; the placentas and fetuses were dissected and weighed. Each placenta was divided in two, one part was fixed in 3.7% phosphate-buffered formalin, and embedded in paraffin, and another part was snap-frozen in liquid nitrogen until determination of arsenical species.

### 2.2. Histopathology Study

Paraffin sections (4 *μ*m thick) were obtained and stained with hematoxylin-eosin (H&E). Morphological alterations were evaluated in the three main areas of the placenta (*decidua basalis*, junction zone, and labyrinth) by optical microscopy (10x and 60x). The analysis of the effects of treatment consisted of recording fibrosis, hemorrhage, and infarcts for the decidua zone; documenting alterations in phagocytosis or abnormal nuclei of cells in the junction zone, and observing vascular congestion and infarcts in the labyrinth zone. The morphological alterations were included in a binary database; qualitative variables were transformed into quantitative data by assigning the number “1” if the alteration was present and the number “0” for the absence of morphological alterations. These data were captured in a spreadsheet, and the percentages of morphologic alterations found in each group were calculated.

### 2.3. Immunohistochemical Analysis (IHC)

The IHC technique was carried out according to the protocol implemented and described by our research group [30, 31] with the following modifications: after blocking (PBS pH 7.4, containing 10% nonfat milk), the slides were incubated separately for 1 hr at 37°C with the corresponding polyclonal goat anti-human antiserum GLUT1, GLUT3, or GLUT4 (1 : 750 dilution in PBS at pH 7.4, containing 1% non-fat milk). The slides were washed and exposed to the secondary (affinity-purified biotinylated rabbit anti-goat IgG) antibody for 1 hr at room temperature. The signal was detected using avidin-peroxidase and freshly prepared diaminobenzidine substrate. Stained slides were dehydrated for permanent cover slipping with Entellan resin. To prevent variability, all of the samples were processed on the same day. Paraffin sections of heart and testis from an unexposed rat were used as controls for heterologous recognition of the GLUT1 and GLUT4 transporters and GLUT3 transporter, respectively. Negative controls were obtained by omitting the primary antibody. For immune localization, slides for each GLUT transporter were contrasted with hematoxylin for 10 min before dehydration.

### 2.4. Image Analysis of GLUT1, GLUT3, and GLUT4 Expression

The level of GLUT1, GLUT3, and GLUT4 expression was obtained by optical density as measured in a BX41 Olympus microscope (Olympus Optical Co., Ltd., Mexico) equipped with a Pixera-CCD camera and analyzed with the IMAGE proplus 4.1 software (Media Cibernetics, Silver Spring, Maryland, USA) [30, 31]. Five representative microphotographs of the labyrinth were taken from each placenta at 60x and six measures of optical density (each with a perimeter of 5 *μ*m) were performed for each sample (*n* = 300). After calibrating the microscope, measurements were made with an individual pixel resolution of 175 grey levels. All determinations were made on the same day to reduce calibration or lighting errors. The samples used for image analysis were not counterstained in order to avoid the background signal for hematoxylin.

### 2.5. Determination of Arsenical Species in Placental Tissue

Quantification of arsenical species (iAs, MAs, and DMAs) was performed by hydride generation-cryotrapping-atomic absorption spectrometry (HG-CT-AAS) [32]. Briefly, the placental tissue was homogenized in deionized water (2.25 mL of water per 0.3 g of tissue) using a pestle homogenizer. The homogenate was placed into a conical tube and 3 mL of 2 M phosphoric acid (Ultrex II) was added. The tubes were heated at 95°C for 1.5 hr using a thermoblock and stirred every 15 min with a vortex until the sample disintegrated. Once digested, the samples were cooled to room temperature and neutralized by adding 700 *μ*L of 10.9 M sodium hydroxide. The final cysteine concentration was adjusted to 2% using a 40% cysteine solution. After 70 min, the level of arsenical species was determined by HG-CT-AAS [32]. Total arsenic (tAs) value was calculated as the sum of the iAs, MAs, and DMAs contents in the placenta. The relative percentages of iAs, MAs, and DMAs in placenta were calculated so that the sum of the arsenicals constituted 100%.

### 2.6. Statistical Analysis

The gestational parameters were analyzed as quantitative variables using an ANOVA test; differences between groups were considered significant when *P* ≤ 0.01 by Bonferroni* post hoc* test. The histopathological changes in placental morphology were treated as outcome variables and were analyzed by a Chi square test. An ANOVA with a Bonferroni multiple comparisons post-test was used to evaluate group-specific differences in the placental distribution for tAs and individual metabolite data. Quantitative data are presented as the means ± standard deviation. Data analyses were carried out with the STATA 9.0 program for Windows (Stata Statistical Software, Release 9.0, Stata Corporation, College Station, Texas, USA).

## 3. Results

### 3.1. Gain of Maternal, Fetal, and Placental Weight

In total, 15 females were included in the study. One hundred twenty five placentas and fetuses were obtained; 39 placentas belong to the group that was not exposed to iAs, and 86 belonged to the iAs-exposed groups. The characteristics of females and products are shown in Table 1. No significant differences were detected in the maternal weight gain or in maternal weight at term between the groups exposed to NaAsO_2_ and the nonexposed females (*P* > 0.05). However, maternal weight at term for females from exposed groups was lower than that of nonexposed females. Otherwise, placental weight was significantly decreased in the group exposed to 20 ppm NaAsO_2_ (*P* < 0.01). In case of fetal weight, a significant decrease was observed in exposed group compared with nonexposed (*P* < 0.01). No difference, in fetal weight, was detected between exposed groups. A significant increase in the number of nonviable fetuses was observed in the group exposed to 12 ppm compared with the nonexposed group (*P* < 0.01).

### 3.2. Morphological Alterations in Placental Tissue

**Figure 1:**
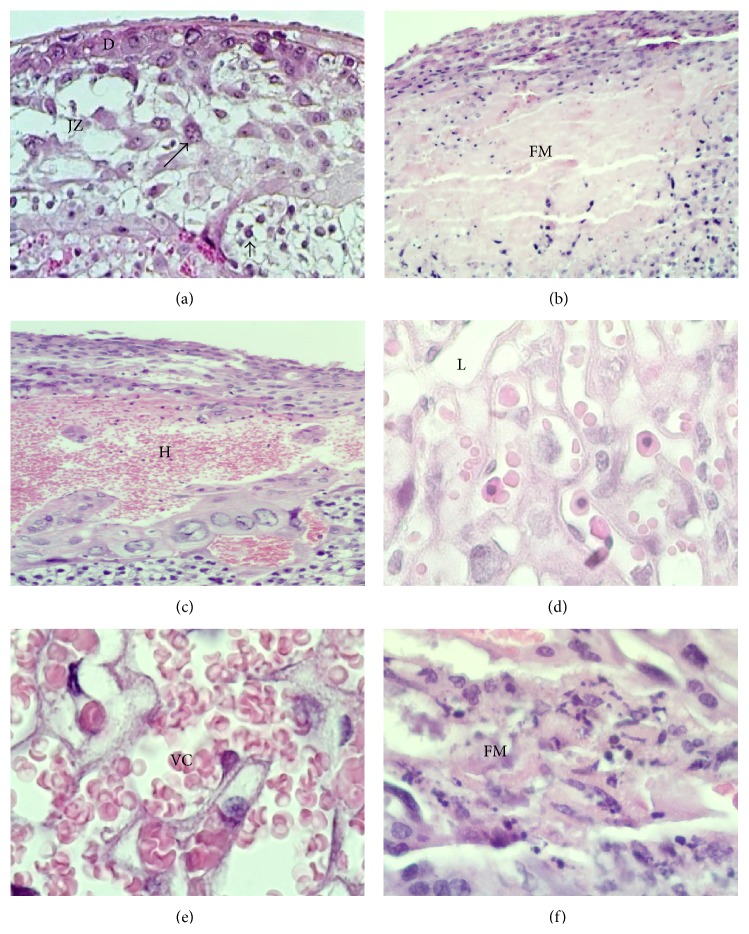
Morphologic alterations found in placentas from female mice exposed to NaAsO_2_ in water. Microphotographs show the decidua (D), junction zone (JZ), and labyrinth (L) zones of murine placenta. (a) Normal morphology of murine placenta and the trophoblast giant cells (arrows) and clusters of glycogen trophoblast cells (arrowheads) are shown in JZ and near to L. Magnification 10x. (b) Infarct and deposits of fibrinoid material (FM) present in JZ and L. (c) Hemorrhagic lesion located in D zone found in placenta from a NaAsO_2_ exposed female. Magnification 10x. (d) Normal appearance of the L zone present in placenta from a nonexposed female. Magnification 60x. (e) Vascular congestion (VC) located in L zone present in the placenta from a NaAsO_2_ exposed female. Magnification 60x. (f) Magnification of fibrinoid material (FM) in the placenta from a female exposed to NaAsO_2_. Magnification 60x. H&E stain.

The morphological analysis of placentas from nonexposed females showed normal cellular architecture (Figure 1(a)); no microscopic alterations were observed in the labyrinth (Figure 1(d)). However, fibrosis and hemorrhagic processes were observed in the decidua basalis area. In placentas from NaAsO_2_ exposed females, it was common to detect hemorrhagic zones (Figure 1(c)) and infarct lesions in decidua basalis, where the cells were replaced by fibrinoid material (Figure 1(b)). In the labyrinth zone, vascular congestion was evident (Figure 1(e)) in placentas from females in the groups exposed to any concentration of NaAsO_2_. Microinfarction with cellular infiltration was detected in the labyrinth in one placenta from a NaAsO_2_ exposed female (Figure 1(f)).

To analyze the differences in placental histological findings among groups, the percentages obtained for each alteration were compared. As shown in Table 2, a significant increase in infarcts in the junction zone was detected in placentas from NaAsO_2_ exposed females, as well as vascular congestion in the labyrinth zone.

### 3.3. Immunelocalization of GLUT1, GLUT3, and GLUT4 in Murine Placenta

**Figure 2:**
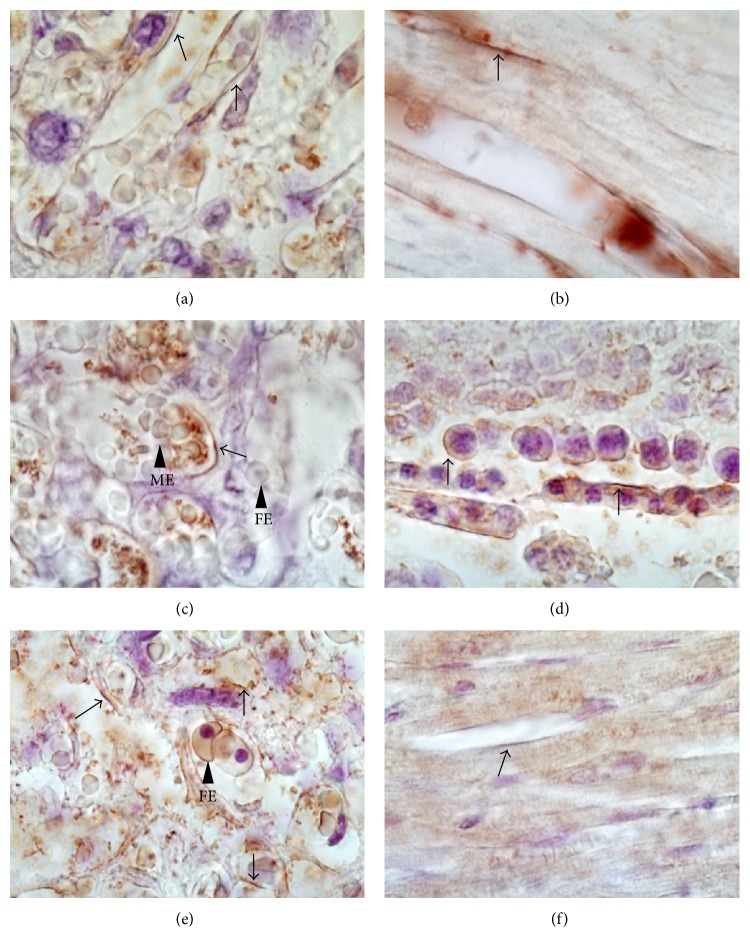
Immunelocalization of glucose transporters in placenta of female mice. The photomicrographs represent immunelocalization of GLUT transporters in placenta from nonexposed females. Samples were counterstained with hematoxylin after an immunohistochemical procedure. (a), (c), and (e) show a positive signal for GLUT1, 3, and 4, respectively, (arrows) in mouse placenta. GLUT1 was identified on both sides of the maternal-fetal interface while GLUT3 was localized mainly in the fetal endothelium. GLUT4 was detected in both syncytiotrophoblast and stromal cells. Positive controls were mounted in rat tissues: (b) heart for GLUT1; (d) testis for GLUT3; and (f) heart for GLUT4. The signal for GLUT1 and GLUT4 was localized on myocytes sarcoplasm and GLUT3 in cytosol of Sertoli cells. ME: maternal erythrocyte; FE: fetal erythrocyte. Magnification 60x.

GLUT1, GLUT3, and GLUT4 were expressed in the labyrinth (Figure 2); in the case of GLUT1, protein expression was identified on both sides of the maternal-fetal interface, mainly in the brush border of the syncytiotrophoblast layer (Figure 2(a)) and weakly in the fetal endothelium. In the case of GLUT3, protein expression was localized mainly in syncytiotrophoblast layer (Figure 2(c)). Finally, GLUT4 expression showed the same pattern as that observed for GLUT1; the protein was expressed on both sides of the maternal-fetal interface (Figure 2(e)). No changes in the localization of GLUT transporters were detected in placentas from females exposed to NaAsO_2_ (data not shown). The controls for antibody heterologous recognition using rat heart (GLUT1; Figure 2(b) and GLUT4; Figure 2(f)) and rat testis (GLUT3; Figure 2(d)) showed strong signal for each protein.

### 3.4. Effect of NaAsO_2_ Exposure on GLUT1, GLUT3, and GLUT4 Expression

**Figure 3:**
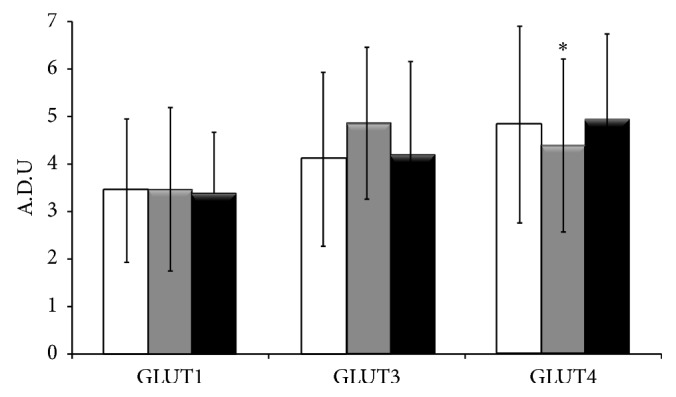
Expression of GLUT1, 3, and 4 in placenta from female mice exposed during gestation to NaAsO_2_ through water. Each bar indicates the mean ± s.d. of arbitrary units of optical density (ADU) obtained for each transporter (*n* = 300 for each protein), according to NaAsO_2_ water concentration: White, 0 ppm; Gray, 12 ppm; and Black, 20 ppm. Comparisons among groups were performed using the Wilcoxon range test; ^∗^ *P* < 0.05.

To analyze the expression of GLUT transporters among groups, image analysis was performed using the methodology previously reported by our research group [30, 31]. The expression of GLUT1 showed no difference between placentas in female controls and those that were exposed to iAs (Figure 3). Although a nonsignificant increase was detected in GLUT3 expression in placentas from female mice exposed to 12 ppm of NaAsO_2_, the expression of this transporter was quite similar in placentas from females exposed to 20 ppm and in nonexposed females.

The expression of GLUT4 was significantly lower (*P* < 0.05) in placentas exposed to 12 ppm of NaAsO_2_ compared with the expression in placentas from control females and those exposed to 20 ppm iAs (Figure 3).

When iAs and its metabolites were quantified in placenta samples (Table 3), higher concentrations of iAs, MAs, and DMAs were found in placentas from exposed females than in those from control mice. The main arsenical species found in placental tissue was DMAs, which agrees with the results reported by Devesa et al. (2006). However, we did not find a significant difference in iAs or in arsenical species concentrations relative to the dose used for iAs exposure. This may be, in part, due to significantly lower water consumption by mice in the 20 ppm as compared to the 12 ppm group. Similar results were observed in Paul et al. 2007 where control mice consumed an average of 5.0 mL of water per day (mL/d). Mice in the 25 ppm and 50 ppm group consumed significantly less water: 3.8 mL and 2.5 mL per day, respectively [33].

## 4. Discussion

In the present study, we used an environmentally relevant concentration of NaAsO_2_. It is recognized that mice metabolize iAs and clear iAs metabolites from tissues more efficiently than humans and that significantly higher exposure levels or longer exposure times are needed in mice to produce symptoms of chronic As toxicity found in humans [33]. Prenatal exposure to NaAsO_2_ in Balb/c strain mice can cause maternal toxicity, which presents as a decrease in body weight of exposed females relative to nonexposed mice [34]. In our study, no maternal toxicity was detected as maternal weight showed no significant difference from the nonexposed group. Although the existing studies are highly diverse, a nonsignificant decrease in body weight in mice exposed to iAs has been reported by another groups [24, 35, 36]. As a result of prenatal exposure, fetuses from exposed litters showed lower body weights compared with fetuses from nonexposed litters, and although fetuses from exposed females were not measured, some of them were smaller than those from nonexposed litters (data not shown). In addition, decrease in placental weight in As exposed females could be due to an impairment in placental vasculogenesis [36], reduction of cytotrophoblastic plugging, and syncytium formation caused by oxidative stress which could generate placental pathology and/or preeclampsia [37–39]. These results suggest that arsenic exposure may induce placental tissue damage and compromise the supply of nutrients, oxygen, hormones, and other growth factors that are necessary for fetal welfare.

The histopathological study revealed morphological changes in placental tissue such as fibrosis, hemorrhage, infarcts, and vascular congestion. These results are consistent with those reported by Levario-Carrillo et al. (2004), who evaluated the effect of methyl parathion, an organophosphate pesticide, on placental morphology in pregnant rats and reported large areas of fibrosis and hemorrhage in decidua basalis as a consequence of exposure. The authors suggested that congestion detected in the labyrinth might act as a compensatory mechanism to dilute the toxic agent and replace the dead cells [40]. However, vascular congestion could be related with deficiencies in vasculogenesis as was reported by Paul et al. [35]. The presence of fibrosis in the junction zone has been suggested as a normal indicator of placental aging [41], and the fibrinoid material found in placentas with infarct lesions suggests a remodeling mechanism to replace the damaged tissue. Considering these findings, we suggest the existence of a general mechanism whereby the placenta responds to aggression regardless of the toxic agent (arsenic, lead, methyl parathion, etc.). In addition, histological alterations found in placentas from exposed females were not severe enough to explain the diminution in placental and fetal weight, which underscores the need to analyze these changes at the molecular level.

The placental expression of GLUT1, GLUT3, and GLUT4 has been reported previously [15, 42], and our findings are consistent with these results. GLUT1 is a ubiquitous isoform expressed in almost all of the tissues examined [18]. The GLUT1 transporter was located in both the junction zone and the labyrinth. Expression in the junction zone could be necessary to satisfy the metabolic demands of the placenta, whereas its expression in the labyrinth could be involved in the maternal-fetal transfer of glucose [42]. The specific expression of GLUT3 in the labyrinth suggests that this transporter is more important for the regulation of glucose transport than GLUT1. GLUT4 expression was localized by Xing et al. (1998) in the stromal cells of human and rodent term placentas using indirect IHC [15]. Nevertheless, we identified the expression in both syncytiotrophoblast and stromal cells. GLUT4 is already synthesized and maintained in subplasmalemmal vesicles. It is primarily responsible for the rapid upregulation of transport activity observed in some tissues (fat and muscle) in response to insulin, which is supplemented by a slower phase of transcription and translation [18]. In the placenta, the syncytial GLUT4 could help improve glucose transport in response to any insulin stimulus (maternal or fetal).

Instead, we observed an increase in the expression of GLUT3 in placentas exposed to 12 ppm of NaAsO_2_ (although the difference was not statistically significant). These results are consistent with those reported by Boileau et al. (1995), who analyzed the expression of GLUT1 and GLUT3 in placentas from diabetic rats and demonstrated an increase in GLUT3 expression, whereas the expression of GLUT1 remained unchanged [43]. Considering this evidence, we suggest that exposure to NaAsO_2_ induces a state of maternal hyperglycemia in which GLUT3 seems to be more sensitive than GLUT1 to blood glucose levels. Additionally, the expression of the placental GLUT4 transporter was decreased in the group exposed to 12 ppm. Decrease of GLUT4 is related to a mechanism to protect the fetus from high levels of maternal glucose [44]. Although we did not determine glucose concentrations in the blood of females during As exposure, hyperglycemia, high levels of insulin, and impaired glucose tolerance in pregnant mice exposed to As have been reported [24, 35]. Alterations in the transfer of glucose are common in many abnormalities of fetal growth (fetal macrosomia and intrauterine growth restriction), and this relationship could explain the adverse pregnancy outcomes associated with iAs exposure. However, in the group exposed to 20 ppm this regulation does not seem to occur because expression did not differ from the control group. These differences are most likely related to the fact that females exposed to this concentration consumed a lower volume of water, as was previously described by Paul et al. [35].

On the other hand, studies performed in a* S. cerevisiae* model have demonstrated that GLUT1 and GLUT4 participate in the uptake of arsenite and MAs, by a different pathway used for glucose, which might contribute to As toxicity [45–47]. In addition, some studies show that glucose uptake can be inhibited by As, even with high levels of insulin [46, 48], these findings suggest that iAs exposure during pregnancy could impair placental capacity for nutrient transfer resulting in pregnancy complications and/or restricted fetal growth [17].

To our knowledge, this is the first report concerning the expression of GLUT4 in placentas from mice exposed to low levels of NaAsO_2_, and our findings are consistent with the mechanism proposed by Paul et al. (2007) to explain the diabetogenic potential of As. Paul et al. (2007) suggest that arsenical trivalent species disrupt the activity of (3-phosphoinositide-dependent) kinases 1 and 2, blocking the intracellular signaling cascade triggered by the insulin receptor and resulting in the blockage of GLUT4 translocation from endosomal vesicles to the plasma membrane, which consequently decreases GLUT4 expression in cells [35]. In our study, the decrease in GLUT4 expression in placentas might explain the low fetal weight observed in the exposed groups. However, more research is required to analyze whether the signaling mechanism of GLUT4 is similar to that reported in adipocyte or muscle cells.

These results are important as they provide evidence that, in pregnant women, even low concentrations of iAs in water may damage the placenta and the fetus, not only affecting its survival* in utero* but also modifying the expression of its genes and increasing the risk of disease in the future.

## Figures and Tables

**Table 1:** Reproductive parameters in animals according to the NaAsO_2_ concentration in water.

Treated group	Concentration of NaAsO_2_ in water (ppm)		
	0 (*n* = 5) $\overset{-}{X}$ ± SD	12 (*n* = 5) $\overset{-}{X}$ ± SD	20 (*n* = 5) $\overset{-}{X}$ ± SD
Increase in body weight (g)^a^	15.36 ± 4.05	7.18 ± 6.02	7.62 ± 5.76
Maternal weight (g)	42.62 ± 3.55	33.8 ± 7.28	34.58 ± 6.69
Placental weight (g)	0.169 ± 0.02^a^	0.154 ± 0.02^a^	0.147 ± 0.02^b^
Fetal weight (g)	0.651 ± 0.09^a^	0.611 ± 0.10^b^	0.528 ± 0.10^b^
Number of viable fetuses/litter	7.6 ± 3.04	7.8 ± 3.5	9.2 ± 2.04
Number of nonviable fetuses/litter	0.2 ± 0.44^a^	1.8 ± 1.09^b^	1.2 ± 0.83^a^

$\overset{-}{X}$  ± SD, mean ± s.d. of each group; *n* = number of females in group.

Different superscript indicate statistical difference *P* < 0.01, by Bonferroni *post  hoc* test.

**Table 2:** Histological findings in mice placentas according to the NaAsO_2_ concentration.

	Concentration of NaAsO_2_ in water (ppm)		
	0	12	20
	*n* (%)	*n* (%)	*n* (%)
Decidua			
Fibrosis	41 (100)	25 (100)	46 (100)
Hemorrhage	29 (71)	18 (72)	40 (87)
Junction zone			
Infarct	24 (58)	20 (80)	39 (85)^∗^
Phagocytosis in TGC	41 (100)	25 (100)	46 (100)
Abnormal nucleus	0	0	0
Degenerative GC	41 (100)	25 (100)	46 (100)
Labyrinth			
Infarct	0	0	1 (2.2)
Vascular congestion	27 (66)	25 (100)^∗^	45 (98)^∗^

*n*  = number of placentas where findings were detected by microscopic observation.

TGC: trophoblast giant cells; GC: glycogenic cells.

^∗^ *P* < 0.01 between NaAsO_2_ concentrations, by Chi square analysis.

**Table 3:** Concentration and relative proportion of iAs and methylated species in placental tissue according to NaAsO_2_ concentration.

Arsenical	Arsenite concentration in drinking water (ppm)					
	0		12		20	
	ng As/g of tissue^a^	%	ng As/g of tissue^a^	%	ng As/g of tissue^a^	%
iAs	1.85 ± 0.05	100	7.425 ± 3.31	21	8.573 ± 4.23	25
MAs	UD	0	1.945 ± 0.54	6	1.592 ± 0.49	5
DMAs	UD	0	25.55 ± 3.87	73	24.21 ± 4.84	70
tAs	1.85 ± 0.05	100	34.92 ± 5.56^∗^	100	34.38 ± 8.69^∗^	100

^a^mean ± s.d.; % of arsenical species in relation to total arsenic (tAs).

UD: undetected concentration.

^∗^Difference between exposed versus control group; *P* < 0.01, one way ANOVA test.

## Abbreviations

As: Arsenic
DMAs: Dimethyl arsenic
GLUT: Glucose transporter
(HG-CT-AAS): Hydride generation-cryotrapping-atomic absorption spectrometry
iAs: Inorganic arsenic
IHC: Immunohistochemical analysis
MAs: Methyl arsenic
NaAsO_2_: Sodium arsenite
tAs: Total arsenic.
